# A distinct Golgi-targeting mechanism of dGM130 in *Drosophila* neurons

**DOI:** 10.3389/fnmol.2023.1206219

**Published:** 2023-06-02

**Authors:** Guo Cheng, Jin Chang, Hui Gong, Wei Zhou

**Affiliations:** ^1^Wuhan National Laboratory for Optoelectronics, Britton Chance Center for Biomedical Photonics, Huazhong University of Science and Technology, Wuhan, Hubei, China; ^2^Research Unit of Multimodal Cross Scale Neural Signal Detection and Imaging, Chinese Academy of Medical Sciences, HUST-Suzhou Institute for Brainsmatics, JITRI, Suzhou, China

**Keywords:** GM130, Golgi apparatus, neuronal polarity, dendrites, Golgi-targeting domains

## Abstract

GM130 is a matrix protein that is conserved in metazoans and involved in the architecture of the Golgi apparatus. In neurons, Golgi apparatus and dendritic Golgi outposts (GOs) have different compartmental organizations, and GM130 localization is present in both, indicating that GM130 has a unique Golgi-targeting mechanism. Here, we investigated the Golgi-targeting mechanism of the GM130 homologue, dGM130, using *in vivo* imaging of *Drosophila* dendritic arborization (da) neurons. The results showed that two independent Golgi-targeting domains (GTDs) with different Golgi localization characteristics in dGM130, together determined the precise localization of dGM130 in both the soma and dendrites. GTD1, covering the first coiled-coil region, preferentially targeted to somal Golgi rather than GOs; whereas GTD2, containing the second coiled-coil region and C-terminus, dynamically targeted to Golgi in both soma and dendrites. These findings suggest that there are two distinct mechanisms by which dGM130 targets to the Golgi apparatus and GOs, underlying the structural differences between them, and further provides new insights into the formation of neuronal polarity.

## 1. Introduction

The Golgi apparatus (Golgi) is the hub of the secretory pathway in eukaryotic cells. The Golgi usually forms a stacked cisternal structure, which is polarized into *cis-, medial- and trans-*sides. In vertebrate Golgi, these stacks can be further linked by ribbons (Klumperman, 2011). Via this polarized structure, newly synthesized proteins and lipids are transported to the *cis*-Golgi and then delivered to their subcellular destinations from the *trans*-Golgi after post-translational modifications (Stalder and Gershlick, 2020).

Golgi matrix protein 130 (GM130) was the first matrix protein found to maintain the organization of Golgi, and is broadly conserved among metazoans (Nakamura et al., 1995; Kondylis et al., 2001). Once synthesized, GM130 is rapidly transported and tightly bound to the Golgi membrane, and subsequently plays multiple roles in the structure and function of the Golgi (Yoshimura et al., 2001; Nakamura, 2010; Huang et al., 2021). Functionally, GM130 is vital for forming the stacked architecture and the ribbon structure of the Golgi (Seemann et al., 2000; Marra et al., 2007; Wei and Seemann, 2010), and for the disassembly and reassembly of the Golgi during mitosis (Lowe et al., 1998). It also provides the dock for tethering transport vesicles at the Golgi membrane (Nakamura et al., 1997) and contributes to regulating the glycosylation (Bhat et al., 2017; Wang et al., 2019) and nucleation of microtubules at the Golgi (Rivero et al., 2009; Wei et al., 2015).

Structurally, GM130 is predicted to have a coiled-coil-rich central portion, with short non-coil N- and C-terminal regions (Nakamura et al., 1995). The N-terminal region of GM130 is positively charged and contains the binding site for p115 (Nakamura et al., 1997). The coiled-coil parts contribute to the rod-like structure of GM130, which extends into the cytoplasm, and also contain the binding sites for Rab proteins (Weide et al., 2001; Sinka et al., 2008). These structural characteristics of the N-terminal and coiled-coil regions allow GM130 to capture and tether transport vesicles (Gillingham and Munro, 2016; Wong et al., 2017). The C-terminus has been considered to contribute to the Golgi binding of GM130 via the interaction with GRASP65. The C-terminus of GM130 is a PDZ ligand motif that can interact with the PDZ domains of GRASP65, and deletion or point mutations disrupting this region can disturb GM130 targeting to Golgi (Nakamura et al., 1997; Barr et al., 1998). Recently, studies showed that GM130 has the ability to recruit GRASP65 to the membrane (Sengupta et al., 2009; Bachert and Linstedt, 2010), and the knockdown or deletion of GRASP65 does not affect the Golgi targeting of GM130 (Puthenveedu et al., 2006; Bekier et al., 2017; Zhang and Seemann, 2021). These results suggest that GM130 may target to the Golgi independent of GRASP65. Therefore, the Golgi-targeting mechanism of GM130 needs to be reexamined.

In neurons, Golgi are found not only in soma, but also in dendrites as Golgi outposts (GOs; De Camilli et al., 1986; Gardiol et al., 1999). Accumulated evidence suggests that the dendritic GOs are functionally and structurally distinct from the somal Golgi. Functionally, GOs can serve as sites for the local secretory (Horton and Ehlers, 2003; Horton et al., 2005; Ye et al., 2007) and acentrosomal microtubule nucleation in dendrites (Ori-McKenney et al., 2012). Structurally, the somal Golgi are organized into large and connected units, whereas dendritic GOs present as tubulo-vesicular structures (Quassollo et al., 2015). In *Drosophila* neurons, different compartments of GOs are even disconnected in dendrites (Zhou et al., 2014; Du et al., 2021). dGM130, the GM130 homologue in *Drosophila*, localizes at both somal Golgi and dendritic GOs, and determines their compartmental organization (Zhou et al., 2014). Thus, it is necessary to elucidate the mechanisms by which dGM130 targets to Golgi in both soma and dendrites.

Here, we investigated the Golgi-targeting mechanism of dGM130 in the dendritic arborization neurons (da) in *Drosophila in vivo*. Based on the structural prediction and the sequence alignment with rat GM130, a series of dGM130 truncations were designed. The contributions of these truncations to Golgi-targeting in soma and dendrites were determined observing of their location in Golgi. Taken together, these results help to identify the Golgi-targeting domains (GTDs) of dGM130, providing opportunities for further exploring the Golgi-targeting mechanisms in soma and dendrites.

## 2. Materials and methods

### 2.1. Structure analysis of dGM130

The amino acid sequence of dGM130 was obtained from NCBI databases (NCBI, Bethesda, MD, United States). The coiled-coil and conserved Golgin domains were identified from InterPro.^1^ The possible higher-order oligomeric structures of coiled-coils were predicted by LOGICOIL (Vincent et al., 2013). Sequence alignments between dGM130 and rat GM130 were generated using BioEdit version 7.0 (Hall, 1999).

### 2.2. Constructs of dGM130 truncations

To generate UAS-EGFP-dGM130 construct, *dGM130* cDNA was amplified from UAS-EBFP-dGM130 (Zhou et al., 2014) and EGFP was fused to the N-terminus of *dGM130*. Then it was transferred into the vector pJFRC-10 × UAS-IVS-CD8-GFP (Addgene plasmid #26214), where the CD8-GFP fragment was replaced between the NotI and XbaI sites.

To generate constructs of dGM130 truncations, appropriate primers for each deletion site were synthesized (Supplementary Table S1) and used in the amplification of truncated cDNA from *dGM130* cDNA. These truncated cDNAs were then transferred into UAS-EGFP-dGM130 to replace *dGM130* cDNA between the BamHI and XbaI sites.

All constructs were verified by sequencing.

### 2.3. Transgenic and used *Drosophila* stocks

To make the transgenic lines of UAS-EGFP-dGM130 and truncations, embryos of PBac{y[+]-attP-3B}VK00033 and P{CaryP}attP40 were collected and microinjected with constructs at 18°C.

The following published stocks were also used in this study: GAL4^19–12^ (Xiang et al., 2010), GAL4 ^109(2)80^ (Gao et al., 1999), UAS-ManII–TagRFPt and *dGM130*^∆23^ (Zhou et al., 2014).

All stocks and crosses were raised in a 25°C incubator with 40–60% humidity.

### 2.4. Confocal microscopy

Confocal imaging was performed using an Olympus FV1000 microscope equipped with a 60 × oil objective lens (NA = 1.42).

EGFP was excited with 488 nm and collected with a 500–530 nm filter; TagRFPt was excited with 543 nm and collected with a BA560IF filter. Snapshot images were captured in the XYZ mode with 0.10 × 0.10 × 1 μm^3^ voxel for dendritic shaft, 0.04 × 0.04 × 0.5 μm^3^ voxel for branch point and 0.02 × 0.02 × 0.5 μm^3^ voxel for soma. In time-lapse imaging of ∆N530 in C3da neurons, images were collected at 10 min intervals for 5 frames. The Z-stack images were processed for maximum projection using Fiji software (NIH, United States) for further analysis (Liu et al., 2022).

### 2.5. Preparation of *Drosophila* larvae for *in vivo* imaging

The procedures for mounting larvae for *in vivo* imaging were as follows: early third-instar larvae were anesthetized with ether, and covered with halocarbon oil 700. Then, the orientation of larvae was adjusted to the dorsal or ventral view. Finally, a coverslip was gently pressed on the high vacuum grease which was placed around the larvae.

In general, the imaging of larvae started once after mounting, and lasted for 30 ~ 40 min to obtain images of da neurons at A4–A6 segments. In the imaging of ∆N530 in all da neurons, in order to record the punctate ∆N530 in neurons, these da neuron groups were sequentially imaged after the larvae were mounted for 30 min, and the imaging lasted for another 30 min.

### 2.6. Imaging analysis

The Golgi-targeting of dGM130 truncations was determined by the colocalization state between the green marker and the ManII–TagRFPt labeled Golgi. The Golgi enrichment of ∆N530 was analyzed by the line profiles of fluorescence intensity obtained from Fiji.

To analyze the fluorescence intensity of dGM130 truncations at the regions of somal Golgi, ROIs were determined from the contour of the Golgi labeled by ManII–TagRFPt, and the mean fluorescence intensities in ROIs were measured for analysis.

To analyze the order among different types of dorsal da neurons in punctate ∆N530 generation, all of the recorded dorsal neuron groups were first classified into groups, with one to seven neurons in a group containing punctate ∆N530 in their somata, and the numbers of each classification were counted; then differences in the punctate ∆N530 generation among neuron types were determined by the proportions of punctate ∆N530 generated in each type of neurons. The order was then finalized by calculating the proportion of each type of neuron in neurons presenting punctate ∆N530 in different group classifications. Neuron groups with none or all of the seven neurons with ∆N530 puncta were excluded from the analysis of the differences in punctate ∆N530 generation among the seven neurons, and the different types of da neurons were distinguished based on their locations and morphological features.

### 2.7. Statistical analysis

Comparative analysis among multiple groups with single or two variables was performed using one-way ANOVA or two-way ANOVA in Prism 8 (GraphPad) software, followed by Tukey or Sidak corrections. Comparison between two groups were used unpaired Student’s t-test. Data presented are in the form of mean ± SEM.

## 3. Results

### 3.1. Prediction of dGM130 structure

To determine the Golgi-targeting region in dGM130, we first analyzed the structure of this coiled-coil protein. dGM130 consists of 795 amino acids. Nine coiled-coil domains and two conserved domains of Golgi proteins were identified from the Pfam database (Supplementary Figure S1A). One coiled-coil domain is located at the N-terminal region, while the other eight are located in the central part, clustered in two regions, namely amino acid residues 138–524 and 638–728. The two conserved Golgin domains are located at residues 274–498 and 605–725, similar to the locations of the coiled-coil regions. The C-terminus is a short non-coil region. Further, we inferred the coiled-coil structures of dGM130 using LOGICOIL, which provided the prediction of a coiled-coil oligomeric state by considering that these structures tend to oligomerize into functional higher-order structures (Vincent et al., 2013). Based on this prediction, two coiled-coil regions were identified in amino acid residues 113–520 and 636–729 (Supplementary Figure S1A). Thus, the dGM130 molecule can be divided into four structural regions: the short N- and C-termini, and two central parts which contain the coiled-coil regions and two conserved Golgin domains.

Besides the coiled-coil structures, we further aligned the amino acid sequences of dGM130 and rat GM130 in the N- and C-termini as well as the proline-rich hinge region between the coiled-coil regions. We found that they are highly conserved in the N-terminal region, and have PDZ ligand motifs at the C-terminus (Supplementary Figure S1B). The corresponding continuous proline sequences, which appear around amino acid residue 441 in rat GM130, were not found in dGM130 (Supplementary Figure S1B). Instead, a histidine-rich region (HRR) was noticed near amino acid residue 550 of dGM130 (Supplementary Figure S1C). This region is rich in basic histidine residues and contains several histidine-proline motifs, suggesting a corresponding hinge region between the coiled-coil regions in dGM130.

In summary, four structural regions of dGM130 were inferred from the structural analysis: the highly conserved N-terminus, a non-coil C-terminus with PDZ ligand motif, and two coiled-coil regions in the central portion, linked by a proline-/histidine-rich region (Supplementary Figure S1D).

### 3.2. Different components of dGM130 are required for Golgi-targeting in soma and dendrites

**Figure 1:**
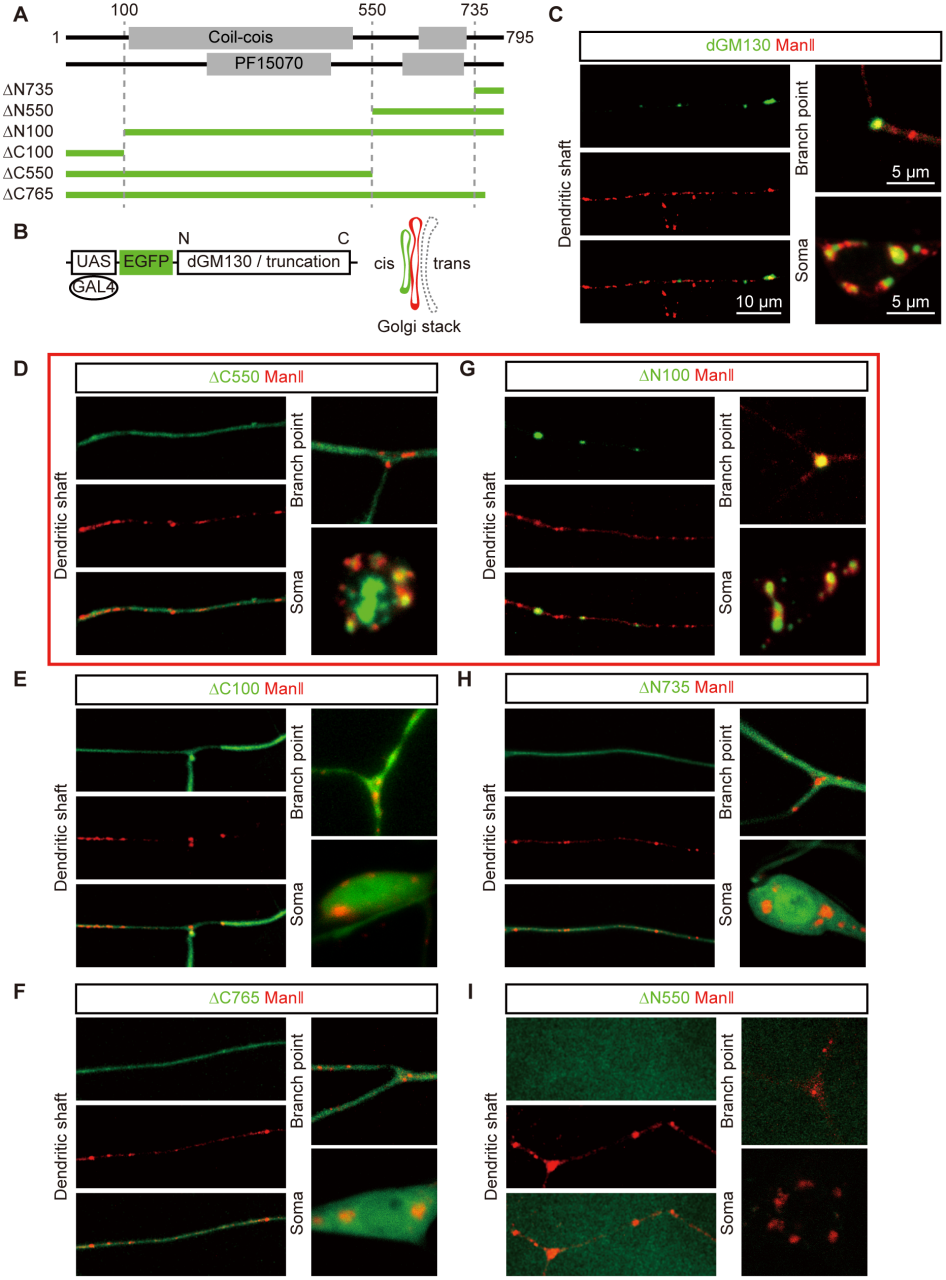
Two dGM130 truncations differentially target to Golgi in soma and dendrites. **(A)** Schematic of the four regions of dGM130 and the N-/C- terminal truncation constructs. Constructs are described according to the position of their first (∆N) or last (∆C) amino acid residue in dGM130. **(B)** Schematic of the strategy for observing the localization of dGM130 and truncations on Golgi *in vivo*. The *medial*-Golgi in red is marked by ManII–TagRFPt. **(C–I)** Representative confocal images show the expression of intact dGM130 and the six truncations (green) together with Golgi marker ManII–TagRFPt (red) in the dendritic shaft, branch points and soma of C3da neurons. Two truncations in red box **(D, G)** showed the ability to bind to Golgi in either soma or both soma and dendrites. No green fluorescence signal could be detected with truncation ∆N550 **(I)**. Scale bars: 10 μm in dendritic shaft, 5 μm in branch point and soma.

To identify the Golgi-targeting regions of dGM130, we designed a series of N-terminal and C-terminal truncations based on the four structural regions predicted above (Figure 1A). To visualize the Golgi and these truncations *in vivo*, fluorescent protein EGFP was tagged at the N-terminal of dGM130 and its truncations, and the Golgi was labeled by Mannosidase II tagged with TagRFPt (ManII–TagRFPt), which is a *medial*-Golgi marker, in the following studies (Figure 1B). The Golgi-targeting states were assessed by analyzing the distributions of EGFP-dGM130 truncations and ManII–TagRFPt, which were specifically expressed in the class III da (C3da) neurons. dGM130 showed different phenotypes in the soma and dendrites. The intact dGM130 proteins were closely juxtaposed with ManII–TagRFPt, forming large units in soma. In contrast, dendritic dGM130 were only colocalized with part of ManII, appearing as puncta (Figure 1C). This is consistent with the morphological features of Golgi reported previously in C3da neurons (Zhou et al., 2014).

Three C-terminal truncations ∆C765, ∆C550 and ∆C100, in which the last one to three structural regions of dGM130 were removed, respectively, showed abnormal distribution in neurons. The ∆C550 truncation, which included the first two regions of dGM130, presented a distinct distribution in the soma and dendrites. It formed large units in the soma, which were closely juxtaposed with ManII, but was diffusely distributed in dendrites (Figure 1D). ∆C100, a further shortening of ∆C550, dispersed completely in both the soma and dendrites (Figure 1E), suggesting that amino acid residues 100–550, which were present in ∆C550 but not ∆C100, are necessary for somal Golgi-targeting. However, ∆C765, which had a deletion of the last 30 amino acid residues at the C-terminus but included the entire region included in ∆C550, was also diffusely distributed in whole neurons (Figure 1F). These results suggested that a complex Golgi-targeting mechanism exists in the neuronal soma and dendrites, such that ∆C550 can only bind to the somal Golgi but not GOs, and the C-terminus of dGM130 is necessary for normal Golgi-targeting.

Among the three N-terminal truncations, ∆N100 with the deletion of the first 100 amino acids, showed punctate distribution in dendrites and presented as large units in the soma (Figure 1G), consistent with the distribution of the intact dGM130, suggesting that the N-terminus does not contribute to the Golgi-targeting. ∆N735, which only retained the last 60 amino acids at the C-terminus, was diffusely distributed in neurons (Figure 1H). Thus, the C-terminus of dGM130 is not by itself sufficient for Golgi-targeting. Unfortunately, we failed to further verify whether adding one more region to the C-terminus would restore the normal Golgi-targeting, as the third N-terminal truncation ∆N550, which was consisted of the last two C-terminal regions, did not show the green fluorescence signal when tagged with EGFP and expressed in C3da neurons (Figure 1I).

Together, these results suggested the possibility that the Golgi-targeting domains (GTDs) located in ∆C550 and the C-terminal region might make distinct contributions to Golgi-targeting in the soma and dendrites.

### 3.3. Golgi-targeting domains1 targets to the Golgi in soma independently

**Figure 2:**
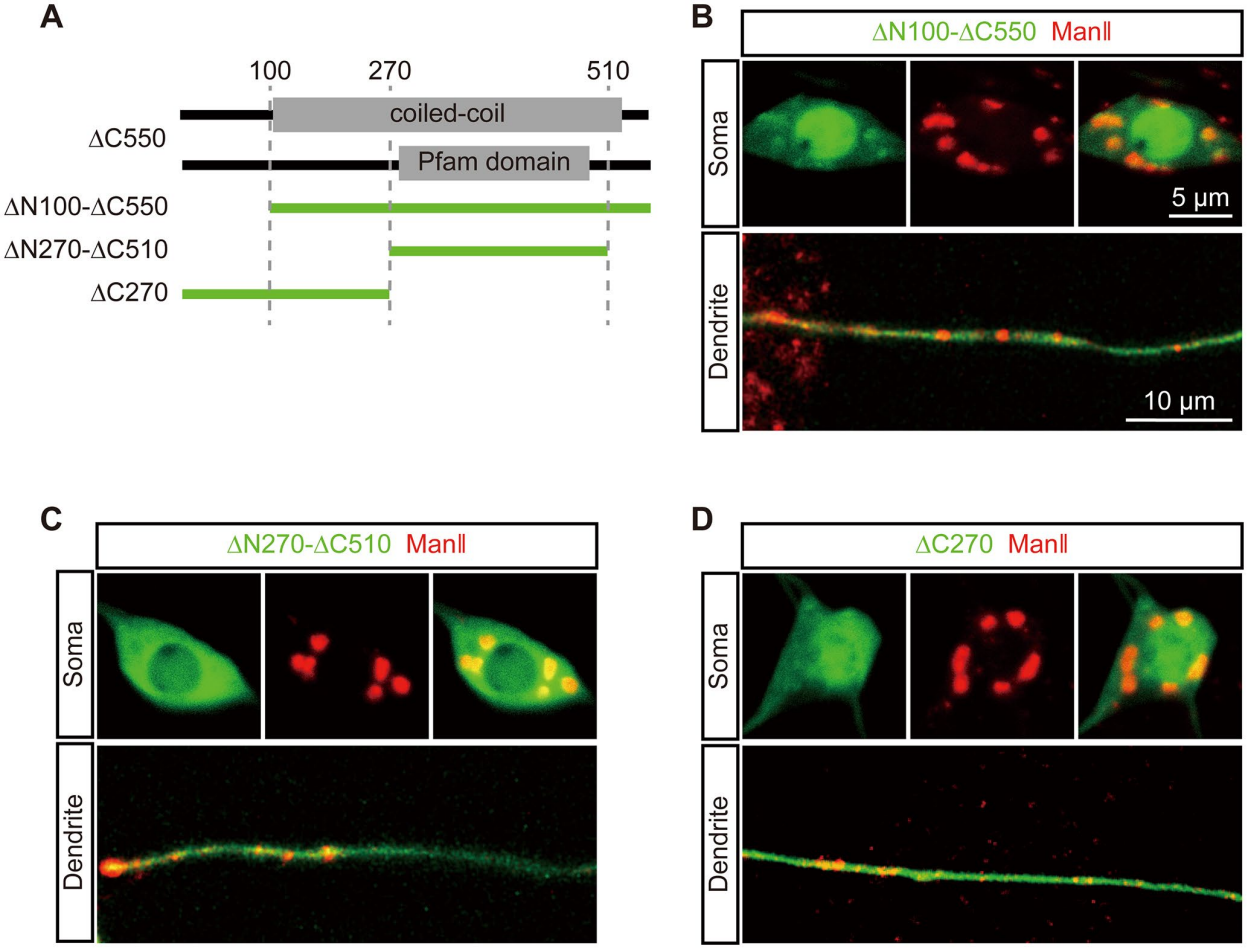
The first coiled-coil region in dGM130 specifically binds to the somal Golgi. **(A)** Upper panels: schematic of the coiled-coil structure and domain in ∆C550. Lower panel: schematic of dGM130 truncations used in this part. **(B–D)** Location of the three EGFP tagged truncations (green) when expressed in C3da neurons. ∆N100-∆C550 in **(B)**, ∆N270-∆C510 in **(C)** and ∆C270 in **(D)**. The Golgi was labeled by ManII–TagRFPt (red). Scale bars: 10 μm in dendrite, 5 μm in soma.

To further pinpoint the GTD in dGM130 for somal Golgi-targeting, we focused on ∆C550. Three precise deletion constructs of ∆C550 were constructed, which were ∆C270, ∆N100-∆C550 and ∆N270-∆C510, considering the conserved domain and coiled-coil region (Figure 2A). *In vivo* imaging showed that only ∆N100-∆C550 had a similar distribution to that of ∆C550: it formed large units and colocalized with ManII in the soma, but diffused in dendrites (Figure 2B). In contrast, neither ∆C270 nor ∆N270-∆C510 truncations had distinctly enriched signal in either somal Golgi or dendritic GOs (Figures 2C,D). Thus, the fragment located at residues 100–550 in dGM130 was identified as the first GTD (called “GTD1”) which was sufficient to target to the somal Golgi. In addition, the GTD1 mainly contains the entire first coiled-coil region (Figure 2A), suggesting that the coiled-coil structure contributes to somal Golgi-targeting.

### 3.4. Second GTD exhibits restricted Golgi-targeting in both soma and dendrites

**Figure 3:**
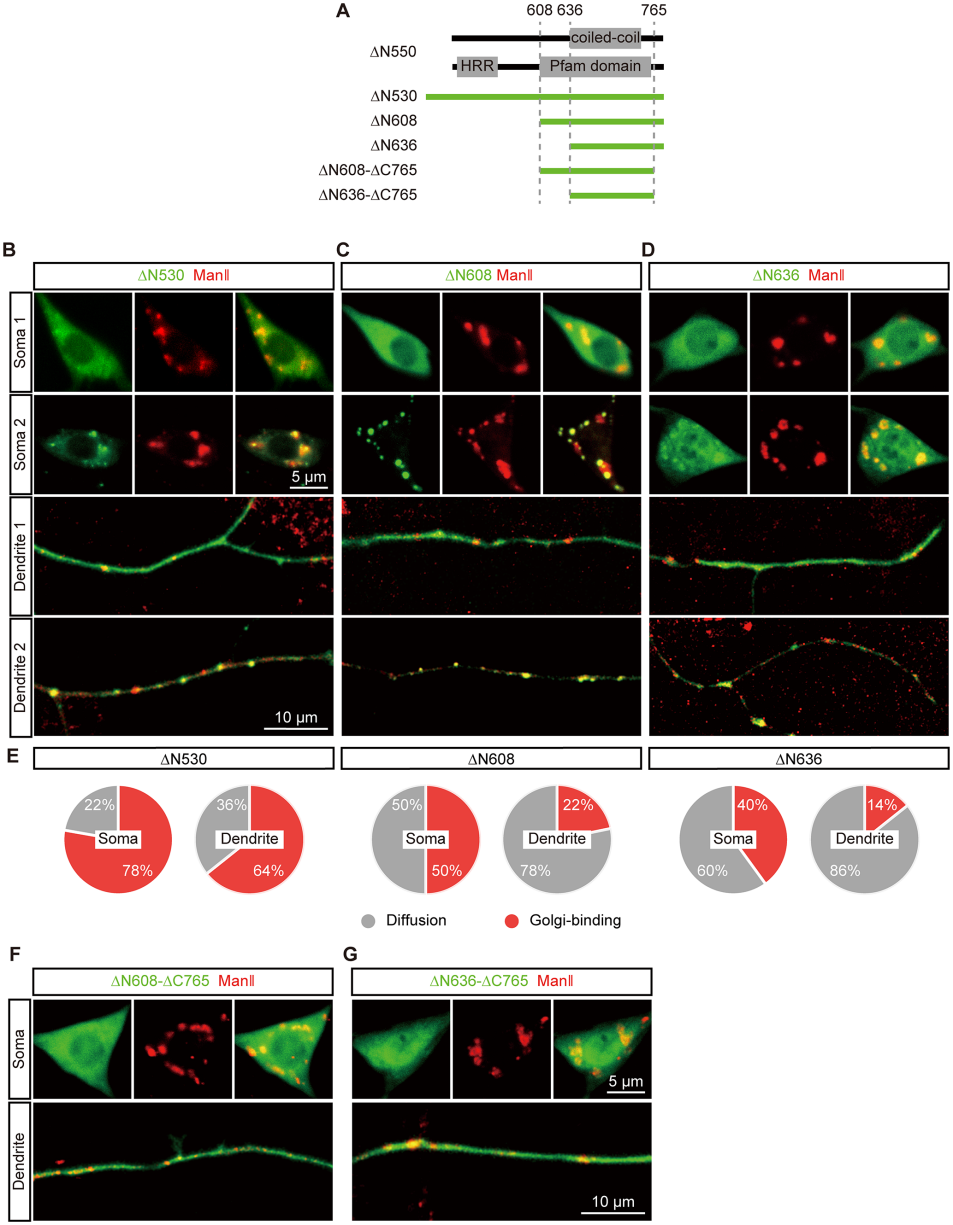
Restricted Golgi-targeting of the C-terminal regions in soma and dendrites. **(A)** Upper panels: schematic of the coiled-coil structure and domain in the C-terminal region. Lower panel: schematic of dGM130 truncations, ∆N530, ∆N608, ∆N636 and ∆N608-∆C765 and ∆N636-∆C765. **(B–D)** Representative images show the diffuse and punctate distribution of ∆N530 **(B)**, ∆N608 **(C)** and ∆N636 **(D)** in soma and dendrites (green) in different neurons. The Golgi was labeled by ManII–TagRFPt (red). **(E)** The pie charts represent the proportion of the two distribution states (punctate and diffuse) of the three C-terminal truncations in the soma and dendrites. **(F,G)** Representative images show the distributions of two C-terminal truncations with the deletion of the C-terminus. ∆N608-∆C765 in **(F)** and ∆N636-∆C765 in **(G)**. Scale bars: 10 μm in dendrite, 5 μm in soma.

Next, it aimed to locate the second GTD (GTD2) in the C-terminal region. We re-designed the ∆N550 construct to extend it to ∆N530 considering that the extended acidic glutamic-acid-rich region might help protein fold correctly, and generated two more truncations, ∆N608 and ∆N636, based on the coiled-coil region and conserved domain (Figure 3A). The expression and Golgi-targeting of the three EGFP tagged truncations were evaluated. All of them could be expressed normally compared to ∆N550 and showed restricted Golgi-targeting in both soma and dendrites. We observed that these truncations showed different types of distribution states in different neurons: in the “punctate” distribution state, truncations were enriched in the Golgi labelled by ManII–TagRFPt and formed puncta, and in the other “diffused” state, truncations were spread throughout the neuron (Figures 3B–D). By further quantitatively analyzing the punctate distribution, we examined the Golgi-targeting of these three truncations in the soma and dendrites, respectively. The proportions of punctate distribution were more in somata rather than in dendrites for all three truncations and increased with the fragment length in both somata and dendrites (Figure 3E). This suggested that the three truncations could each be enriched in both somal Golgi and dendritic GOs, although with a somal Golgi preference, and the extension in sequence promoted this enrichment. Meanwhile, we deleted an additional 30 amino acids at the C-termini of ∆N608 and ∆N636, producing two new truncations, ∆N608–∆C765 and ∆N636–∆C765 (Figure 3A). Neither of them formed puncta (Figures 3F,G), confirming the C-terminus is necessary for dGM130 targeting to Golgi.

Besides, the distribution states and expression levels of the truncations in *dGM130* null neurons were inspected, including ∆N530, ∆C550, ∆C765, ∆C100, and ∆N735. All truncations remained the same distribution states as wild-type neurons (Supplementary Figures S2A–E). And the fluorescence intensities also indicated no significant difference among them when normalized by the fluorescence of co-expressed ManII–TagRFPt (Supplementary Figure S2F).

Taken together, these findings indicated that GTD2 is located in the last two C-terminal regions of dGM130 and exhibits restricted Golgi-targeting in both the soma and dendrites. Upon further comparison with the predicted domains, we found that GTD2 contained the second coiled-coil region and the C-terminus of dGM130, suggesting that this coiled-coil structure contributes to the ability of dGM130 to target to dendritic GOs, as well as somal Golgi.

### 3.5. Diffused GTD2 is gradually enriched in somal Golgi and dendritic GOs

**Figure 4:**
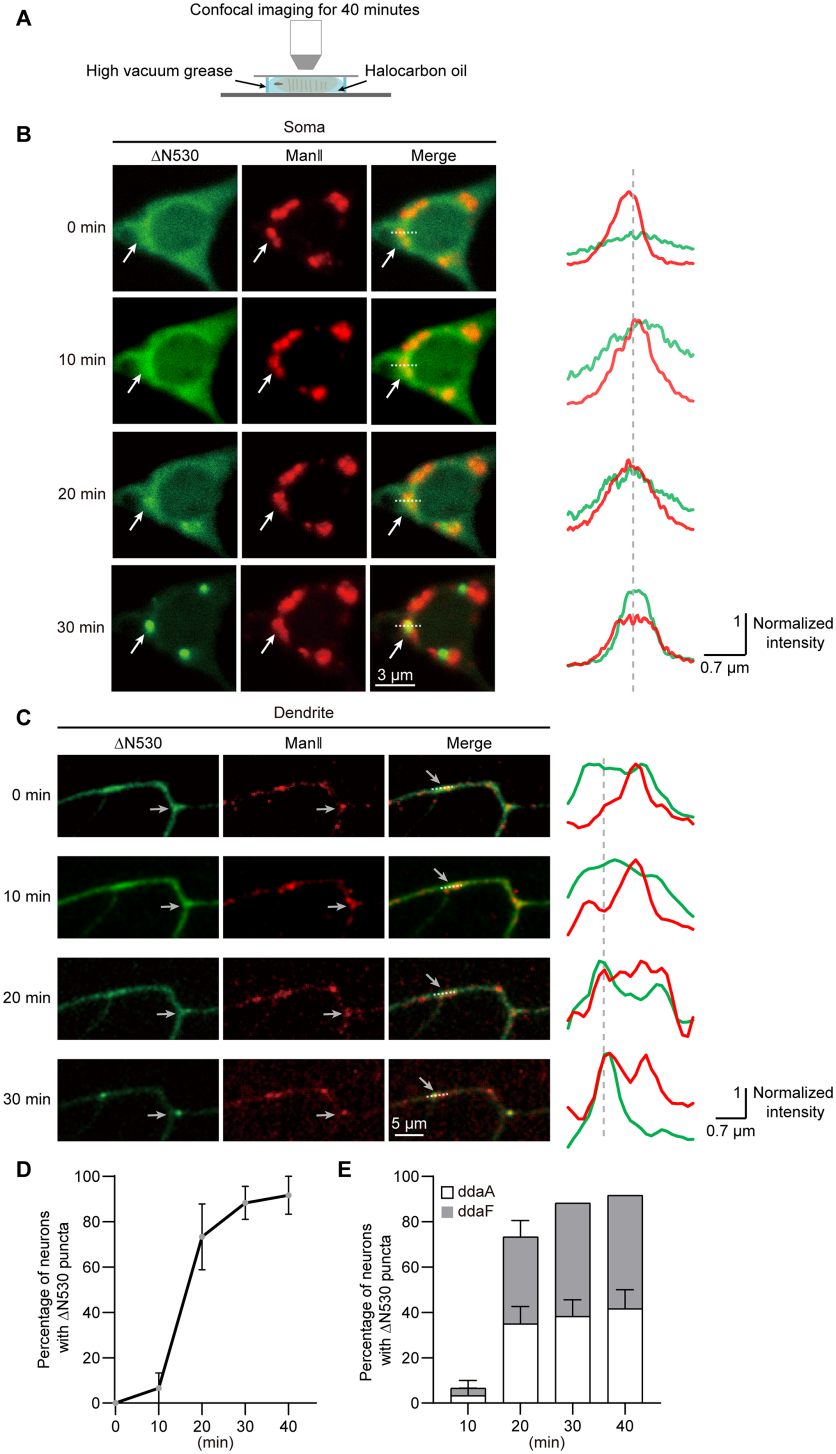
∆N530 is dynamically enriched in Golgi. **(A)** Schematic of the strategy for time-lapse imaging of ∆N530 in *Drosophila* larva. **(B,C)** Representative images in left show the distribution of ∆N530 (green) in soma **(B)** and dendrites **(C)** at three time points. The Golgi was labeled by ManII–TagRFPt (red). Line profiles at right show the normalized fluorescence intensities of EGFP and TagRFPt along the dotted lines at left. The arrows indicate a Golgi unit in soma **(B)** and a dendritic GO **(C)** with the enrichment of ∆N530. **(D)** Quantitation of neurons with Golgi-targeting ∆N530 at different imaging durations. **(E)** Bar chart shows the proportions of the two types of C3da neurons, ddaA and ddaF, that present Golgi-targeting ∆N530 at different imaging durations. For all quantifications, data are shown as mean ± SEM. Unpaired two-sided Student’s *t*-test in **(E)** at each time duration found no significant difference. Scale bars: 3 μm in **(B)**, 5 μm in **(C)**.

To examine how the two distribution states of GTD2 were generated, we conducted confocal time-lapse imaging of C3da neurons that expressed ∆N530 (Figure 4A). We found that ∆N530 progressively condensed from diffuse to punctate in both soma and dendrites, and these ∆N530 puncta persisted once generated (Figures 4B,C). The time-course for Golgi-targeting of ∆N530 was analyzed by quantifying neurons presenting Golgi-targeted ∆N530 over different imaging durations. We found that the ∆N530 targeted to Golgi in 88.3% of neurons within 30 min, and in almost all neurons within 40 min (Figure 4D). In addition, we compared the Golgi-targeting procedures of ∆N530 in two types of C3da neurons, ddaA and ddaF. We found no significant differences between them (Figure 4E), suggesting that the Golgi-targeting of ∆N530 is basically synchronous in the two types of C3da neurons. In short, diffused ∆N530 could progressively target to Golgi and then remain stably enriched there.

As a control, we also checked the distribution states of other truncations with time-lapse imaging, including ∆C550, ∆C100, ΔC765, and ΔN735. The results showed that the distribution states of these truncations remained stable in the soma and dendrites in 40 min (Supplementary Figures S3A–D), indicating that the dynamic Golgi-targeting occurred only in GTD2, among all truncations.

### 3.6. Orderly Golgi-targeting of GTD2 in different classes of da neurons

We further examined the Golgi-targeting of ∆N530 in other types of da neurons to verify whether it is specific for C3da neurons. There are 15 da neurons in per abdominal hemisegment of *Drosophila* larva, which can be classified into four classes based on the complexity of their dendritic branches (Grueber et al., 2002; Supplementary Figure S4A). We expressed ∆N530 in all da neurons driven by Gal4-109(2)80 and then imaged the da neuron groups on the ventral and dorsal sides of the larvae within 30–60 min after being mounted, given that punctated ∆N530 presented in most C3da neurons within 30 min. ∆N530 puncta could be found in all of the four classes of da neurons (Supplementary Figures S4B,C), suggesting the Golgi-targeting of ∆N530 was consistent across these neurons. Then, the distribution of ∆N530 puncta was analyzed on the seven dorsal neurons, including C1da neurons ddaD and ddaE, C2da neuron ddaB, C3da neurons ddaA and ddaF, C4da neuron ddaC and one dorsal multiple dendrite neuron (dmd). Most images presented three or four neurons containing punctate ∆N530 (Supplementary Figure S4D,E). The difference in the proportion of neurons containing punctate ∆N530 was further quantified. Among the four classes of da neurons, the proportions of C3da, C4da, C2da, C1da and dmd neurons decreased successively (Supplementary Figure S4F). Meanwhile, the proportion was also calculated for each image and it was found that punctate ∆N530 appeared in C3da, C4da, C2da and C1da neurons successively, along with an increase in the number of punctated neurons in the image (Supplementary Figure S4G). Thus, these results indicated that ∆N530 could target to Golgi in all da neurons, appearing in C3da, C4da, C2da, and C1da neurons in order.

## 4. Discussion

In this study, we investigated the targeting mechanisms of dGM130 to the somal Golgi and dendritic GOs, which show different compartmental organizations and functions, in *Drosophila* da neurons. We found that two GTDs in dGM130 play different roles in targeting Golgi and GOs, and the integrity of both GTDs is necessary to ensure normal Golgi-targeting in neurons. Of these two GTDs, GTD1 covers only the first coiled-coil region and contributes to somal Golgi-targeting, while GTD2 includes the second coiled-coil region and C-terminus, and it gradually accumulates from a diffuse distribution in the somal Golgi apparatus and dendritic GOs. Our findings demonstrate the distinct Golgi-targeting mechanisms of dGM130 in soma and dendrites, and also highlight the necessity of the coiled-coil structures of dGM130 in Golgi-targeting.

Golgi-targeting domain1 represents a previously unknown Golgi-targeting domain. In mammalian cells, the Golgi targeting of GM130 is achieved via its C-terminus interacting with PDZ domains of GRASP65 (Nakamura et al., 1997; Barr et al., 1998). In *Drosophila*, the PDZ domains are conserved in dGRASP, which is the homologue of GRASP65 (Kondylis et al., 2005), and a PDZ ligand motif also exists at the C-terminus of dGM130 (Jelen et al., 2003; Bachert and Linstedt, 2010). However, the C-terminus, dGM130-∆N735, does not show Golgi-targeting ability. Unexpectedly, the GTD1 in dGM130, which is located at amino acid residues 100–550, targets to the somal Golgi rather than GOs. We therefore propose a Golgi-targeting pathway independent of the C-terminus of dGM130. Interestingly, GM130-∆C983 contains the homologous sequence of GTD1, namely the first three coiled-coil domains, but fails to target the Golgi in HeLa cells (Barr et al., 1998). It is thought to be due to the inclusion of other C-terminal coiled-coil domains, just like the diffuse distribution of dGM130-∆C765.

The structural composition of GTD2 is evolutionarily conserved. GTD2 in dGM130 contains the second coiled-coil region and C-terminus. Its homologous sequence in rat GM130 includes the last three coiled-coil domains and the C-terminus. The homologous rat GM130-∆N690, is also required for Golgi-targeting (Nakamura et al., 1997). This suggests that these two homologues are also functionally conserved in Golgi-targeting. The PDZ ligand motifs at the C-termini of rat GM130 and dGM130 may contribute to the Golgi-targeting of GM130 via the interaction with GRASP65, and the coiled-coil regions provide the oligomeric structural basis for this interaction. However, their time courses for Golgi-targeting are different. GM130-∆N690 can directly and rapidly target to Golgi, like the intact GM130 (Yoshimura et al., 2001). Here, the dynamic Golgi-targeting process of GTD2 was observed, specifically a gradual change from diffuse distribution to Golgi enrichment over a time course ranging from 30 min to more than 1 h, in different classes of da neurons. As the control, the morphology of Golgi marked by ManII was unaffected throughout the process. It provides a model for studying GM130 transport from ER to the Golgi.

The different Golgi-targeting properties of GTD1 and GTD2 are helpful for understanding the different compartmental organizations of Golgi and dendritic GOs. The distinct compartmental organization of GOs has been revealed to be determined by the specific distribution of dGM130 in dendrites (Zhou et al., 2014). In the two GTDs of dGM130, GTD1 targets to somal Golgi rather than GOs, while GTD2 targets to both. Thus, different Golgi-targeting modes of dGM130 appear in the soma and dendrites: dual anchoring at somal Golgi, contrasted with single C-terminal anchoring at GOs. Given the rod-like structure formed by coiled-coils (Ishida et al., 2015), the different anchoring modes of dGM130 may generate different orientations at the Golgi membrane, which can affect the distribution of dGM130 by homotypic tethering (Marra et al., 2007). Dual anchoring mode mediating homotypic tethering at opposing membrane has also been shown for GRASP65, through a mitochondrial relocation assay (Bachert and Linstedt, 2010). These findings suggest that different Golgi-targeting modes of dGM130 underlie the different compartmental organizations of Golgi and GOs.

In addition, the distribution of GTD1 also indicates the difference between the neuronal soma and dendrites. Neurons are highly polarized cells, not only in terms of their morphology and functions, but also in the distribution of lipids and proteins (Horton et al., 2005; Ye et al., 2007; Kennedy and Hanus, 2019). The asymmetric distribution of GTD1 in the soma and dendrites suggests that it may play a role in neuronal polarity. Notably, cell polarity-associated proteins, such as Stk25, YSK1 and RasGRF2, have been found to be partner proteins of GM130 at Golgi membrane (Preisinger et al., 2004; Matsuki et al., 2010; Baschieri et al., 2014). This also suggests that GTD1 specifically targets to the somal Golgi maybe through the interaction with the cell polarity-associated proteins.

Taken together, our findings reveal a distinct Golgi-targeting mechanism of dGM130, with two GTDs performing different targeting modes in the soma and dendrites. This provides a possible reason for the different Golgi compartmental organizations that form in the neuronal soma and dendrites, and could lead to the discovery of new therapies targeting GM130 to fight nervous system diseases.

## Data availability statement

The raw data supporting the conclusions of this article will be made available by the authors, without undue reservation.

## Author contributions

WZ, JC, and HG conceived this project. WZ, JC, and GC designed the experiments and wrote the manuscript. GC conducted the experiments and analyzed the data. All authors contributed to the article and approved the submitted version.

## Funding

This work was supported by the STI2030-Major Projects (2021ZD0201001 to HG), the National Natural Science Foundation of China (61890951 and 31871027 to WZ), and Fundamental Research Funds for the Central Universities (HUST: 2019KFYXMBZ011, 2019KFYXMBZ039, 2018KFYXMPT018, and 2019KFYXMBZ009 to HG), CAMS Innovation Fund for Medical Sciences (CIFMS, 2019-I2M-5-014 to HG) and the director fund of the WNLO.

## Conflict of interest

The authors declare that the research was conducted in the absence of any commercial or financial relationships that could be construed as a potential conflict of interest.

## Publisher’s note

All claims expressed in this article are solely those of the authors and do not necessarily represent those of their affiliated organizations, or those of the publisher, the editors and the reviewers. Any product that may be evaluated in this article, or claim that may be made by its manufacturer, is not guaranteed or endorsed by the publisher.

## Footnotes

^1^ https://www.ebi.ac.uk/interpro/
