# Supplementary material for: A distinct Golgi-targeting mechanism of dGM130 in Drosophila neurons
Source: Front Mol Neurosci. 2023 Jun 2;16:1206219. doi: 10.3389/fnmol.2023.1206219 (PMC10272413; doi:10.3389/fnmol.2023.1206219)
Supplement: Supplementary file 1 [file Data_Sheet_1.pdf]

Supplementary Material

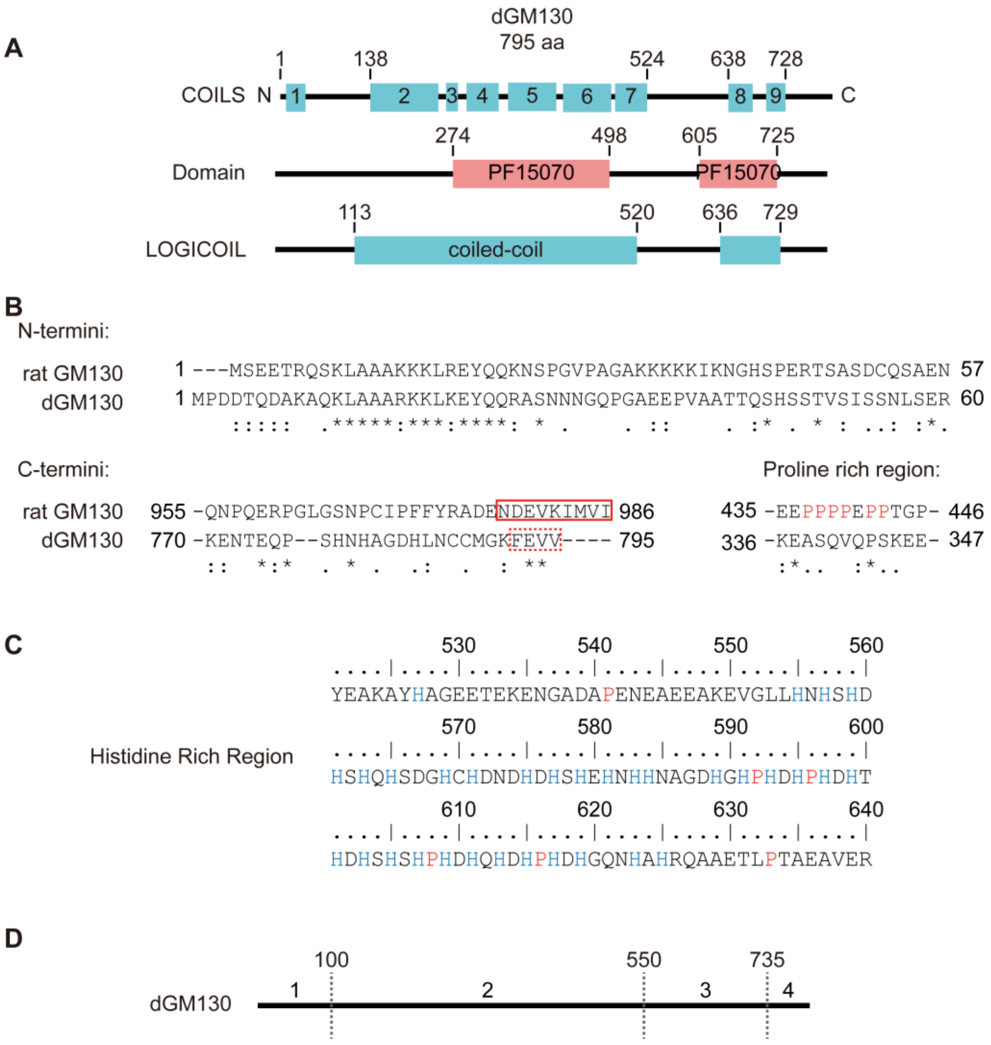

Supplementary Figure 1. The structure prediction of dGM130. **(A)** Schematic representations of the dGM130 structure. The amino acid sequence of dGM130 was analyzed using the Pfam database (top and middle) and LOGICOIL (bottom). The blue boxes indicate the predicted coiled-coil structures and the red ones indicate the conserved Golgin domains. The numbers above the boxes indicate amino acid residues. **(B)** Sequence alignments of the N- / C-termini and the proline-rich region between dGM130 and rat GM130. Asterisks indicate identical residues; colons indicate conserved residues and periods indicate semiconserved residues. The red solid, and dotted boxes at the C-termini indicate the PDZ-ligand motifs in rat GM130 and dGM130, respectively. The continuous proline sequences in rat GM130 are shown in red. **(C)** A histidine-rich region of dGM130 is located at residues 555-625. The histidine residues are shown in blue and the proline residues in red. **(D)** The schematic shows the predicted four regions of dGM130, which are separated at residues 100, 550 and 735.

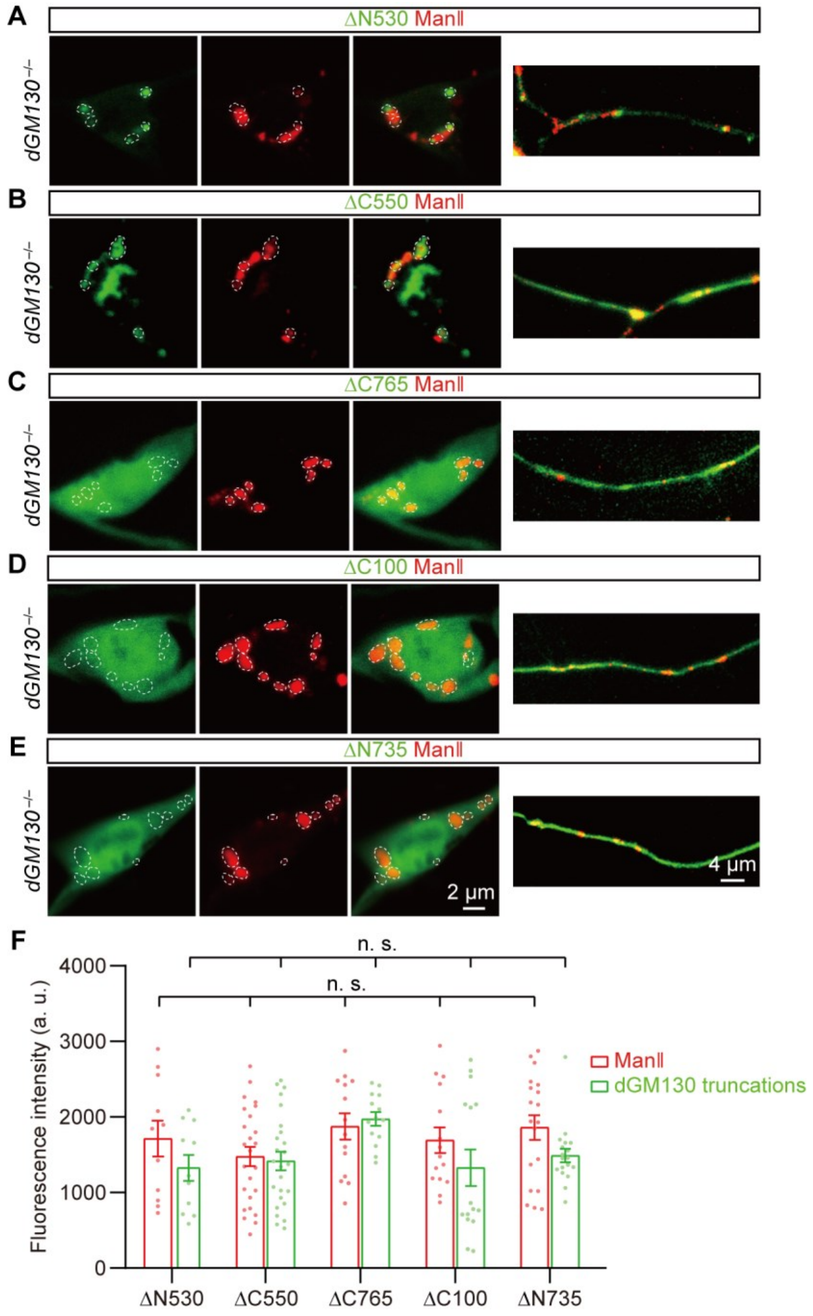

Supplementary Figure 2. The Golgi-targeting states of *dGM130* truncations in *dGM130* null neurons. **(A-E)** Location of EGFP tagged truncations (green) in somata and dendrites when expressed in *dGM130* mutant (*dGM130*$^{-/-}$) neurons. ΔN530 in **(A)**, ΔC550 in **(B)**, ΔC765 in **(C)**, ΔC100 in **(D)** and ΔN735 in **(E)**. The Golgi was labeled by ManII-TagRFPt (red). **(F)** Fluorescence intensities of EGFP tagged truncations and ManII-TagRFPt in the somal Golgi regions, which were indicated in the white circles in **(A-E)**. Data are shown as mean ± SEM. Two-way ANOVA followed by Sidak multiple comparisons test in **(F)**, n. s., not significant. Scale bars: 4 μm in dendrite, 2 μm in soma.

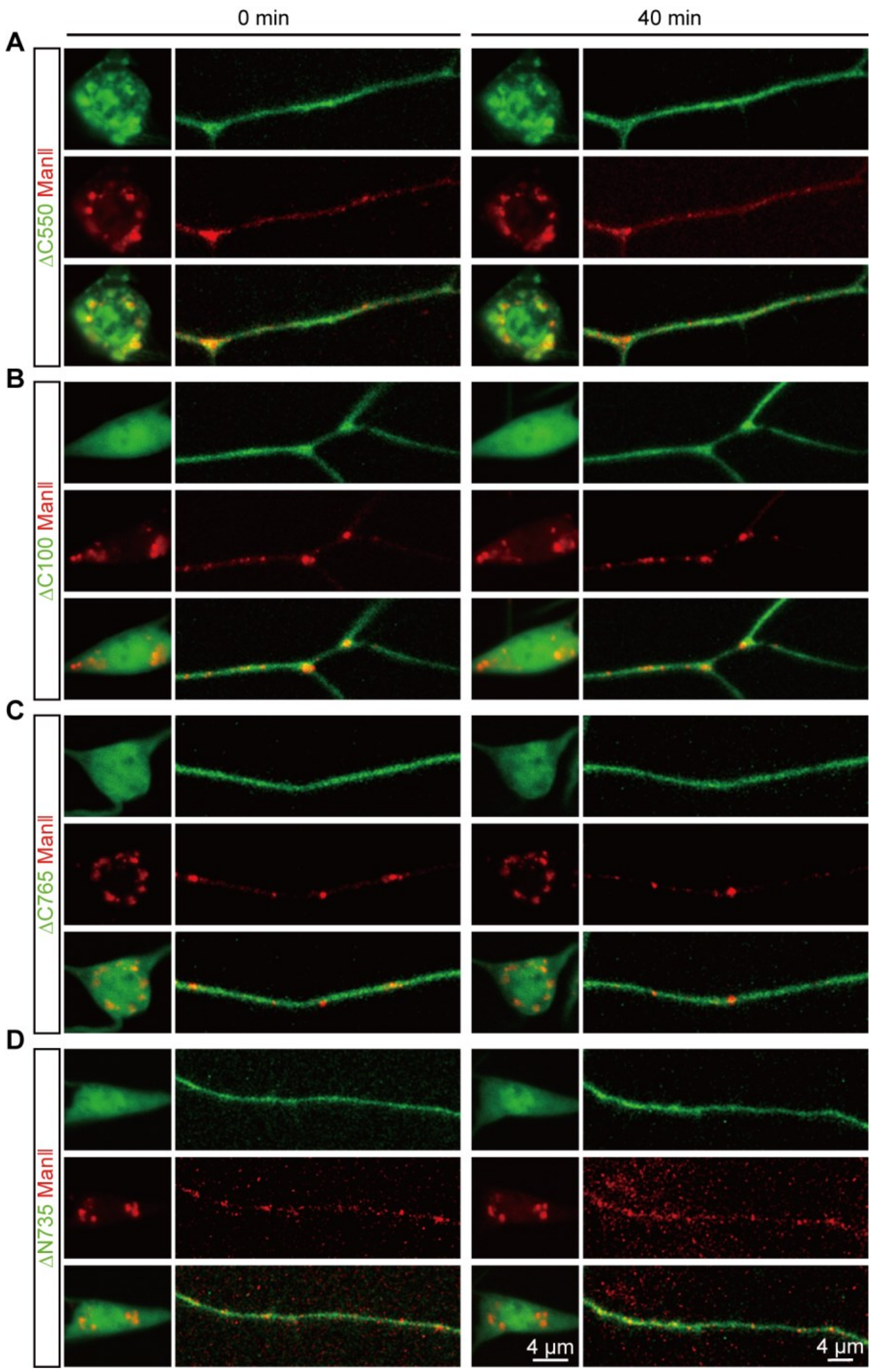

Supplementary Figure 3. The stable distribution states of ΔC550, ΔC100, ΔC765 and ΔN735 in soma and dendrites within 40 minutes imaging. **(A-D)** Representative confocal images show the distribution of dGM130 truncations (green) in the soma and dendrites at the beginning of imaging (0 min, left) and 40 minutes (right). ΔC550 in **(A)**, ΔC100 in **(B)**, ΔC765 in **(C)** and ΔN735 in **(D)**. The Golgi was labeled by ManII-TagRFPt (red). Scale bars: 4 μm.

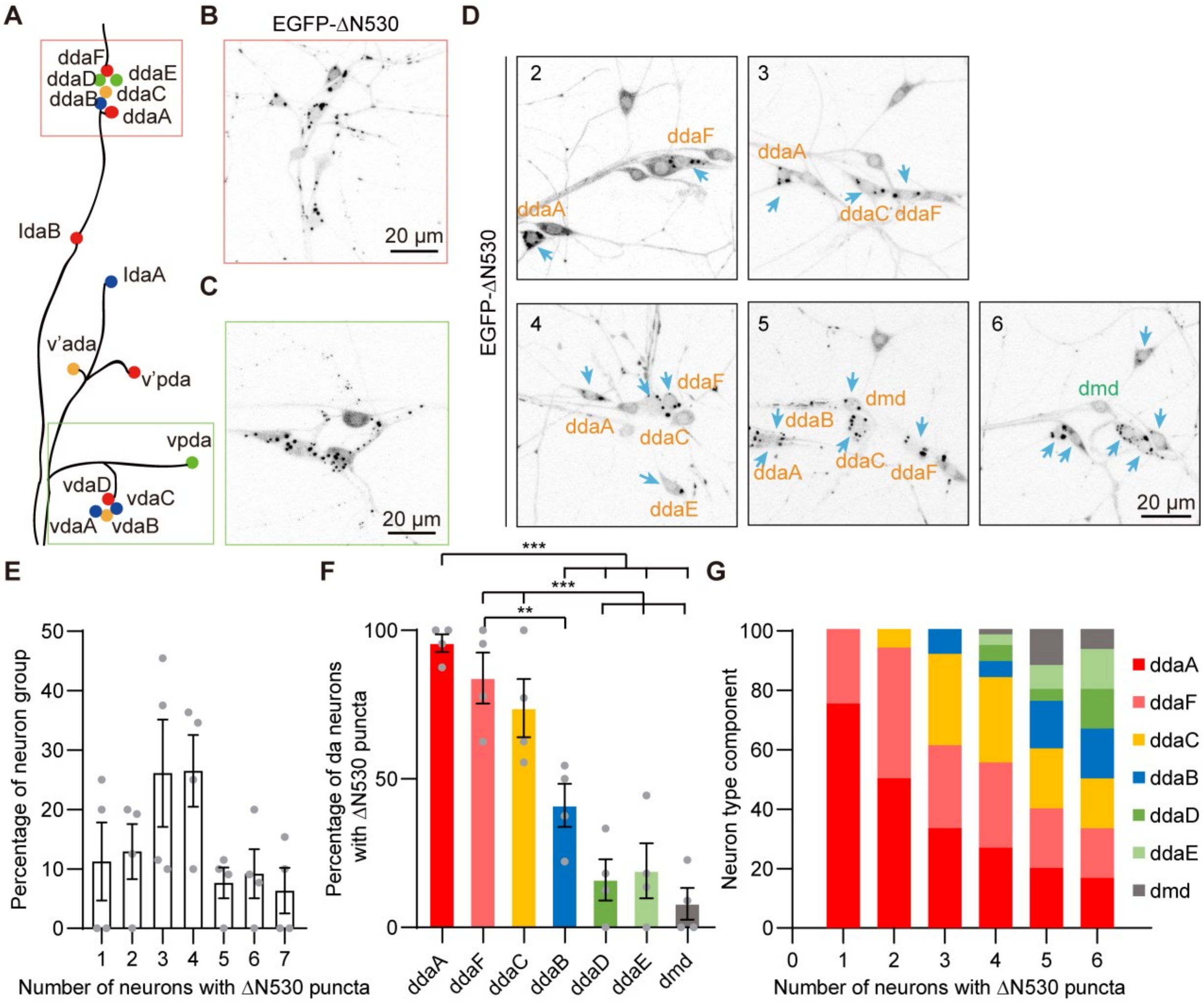

Supplementary Figure 4. Different time courses of ΔN530 enrichment in four classes of da neurons. **(A)** Schematic of abdominal peripheral nerves in a single hemisegment of *Drosophila*. The circles in four colors indicate somata of the four classes of da neurons (green for C1da, blue for C2da, red for C3da, and yellow for C4da). The ventral and dorsal da neuron groups are indicated in green and orange boxes, respectively. **(B-C)** Confocal images show the presence of ΔN530 puncta in dorsal **(B)** and ventral **(C)** da neurons. **(D)** Representative images show the different stages during the enrichment process among the seven neurons. Arrows indicate neurons that present punctate ΔN530. **(E)** Quantification of the number of neurons presenting ΔN530 puncta in each snapshot. **(F)** Quantification of the percentage of neurons with ΔN530 puncta for seven neuronal types. **(G)** Bar chart showing the type components of neurons with ΔN530 puncta in each snapshot, sorted by the number of punctate neurons in a snapshot. Data are the means ± SEM. One-way ANOVA followed by Tukey's multiple comparisons test in **(F)**; $^{**}p < 0.01$, $^{***}p < 0.001$. Scale bars: 20 μm in **(B-D)**.

Supplementary Table 1. constructs and primers used in this study

Constructs	Primers	Sequences of primer
dGM130	GM130-clone-F3	AGCTGTACAAGGGATCCCCCGATGACACGCAGGA TGC
	GM130-N-R	TCCTTCACAAAGATCCTCTAGATTAGACTACCTCG AACTTGCCC
ΔN100	GM130-N100-F	GACGAGCTGTACAAGGGATCCCTGGCAGATGCCT CTAGCTTGC
	GM130-100-R1	TCCTTCACAAAGATCCTCTAGATTACAGTCCACCA TTGTGTTCCG
ΔC100	GM130-clone-F3	AGCTGTACAAGGGATCCCCCGATGACACGCAGGA TGC
	GM130-100-R1	TCCTTCACAAAGATCCTCTAGATTACAGTCCACCA TTGTGTTCCG
ΔN550	GM130-N550-F	GACGAGCTGTACAAGGGATCCGTTGGTCTTTTGCA TAATCATTCGC
	GM130-N-R	TCCTTCACAAAGATCCTCTAGATTAGACTACCTCG AACTTGCCC
ΔC550	GM130-clone-F3	AGCTGTACAAGGGATCCCCCGATGACACGCAGGA TGC
	GM130-550-R1	TCCTTCACAAAGATCCTCTAGATTAAACTTCCTTC GCTTCCTCTGC
ΔN735	GM130-N735-F	GACGAGCTGTACAAGGGATCCGATGCCGCCCAGG CAAAGCAAC
	GM130-N-R	TCCTTCACAAAGATCCTCTAGATTAGACTACCTCG AACTTGCCC
ΔC765	GM130-clone-F3	AGCTGTACAAGGGATCCCCCGATGACACGCAGGA TGC
	GM130-765-R1	TCCTTCACAAAGATCCTCTAGATTATGCCTGGGCG GCATCTGCGG
ΔN530	N530-F	GACGAGCTGTACAAGGGATCCGAGGAAACCGAAA AAGAGAATGGAG
	GM130-N-R	TCCTTCACAAAGATCCTCTAGATTAGACTACCTCG AACTTGCCC
ΔN608	N608-F	GACGAGCTGTACAAGGGATCCCCACACGATCATC AGCATGATC
	GM130-N-R	TCCTTCACAAAGATCCTCTAGATTAGACTACCTCG AACTTGCCC
ΔN636	N636-F	GACGAGCTGTACAAGGGATCCGAGGCCGTGGAAC GCTTGC
	GM130-N-R	TCCTTCACAAAGATCCTCTAGATTAGACTACCTCG AACTTGCCC
ΔC270	GM130-clone-F3	AGCTGTACAAGGGATCCCCCGATGACACGCAGGA TGC
	C270-R	TCCTTCACAAAGATCCTCTAGATTACTGCGTTAGT TGACCCACC

ΔN100-ΔC550	N100-C550-F	GACGAGCTGTACAAGGGATCCGGACTGGCAGATG CCTCTAGC
	N100-C550-R	TCCTTCACAAAGATCCTCTAGATTATTCCTTCGCTT CCTCTGC
ΔN270-ΔC510	N270-C510-F	GACGAGCTGTACAAGGGATCCCAGGACAAGGAGC AGGCAGC
	N270-C510-R	TCCTTCACAAAGATCCTCTAGATTACATTAACTGC TGACGTTGTAG
ΔN608-ΔC765	N608-C765-F1	TGGACGAGCTGTACAAGGGATCCCCACACGATCA TCAGCATGATCATCC
	C765-R	TCCTTCACAAAGATCCTCTAGATTAAATGTCCTGT ATCTTGTTGATG
ΔN636-ΔC765	N636-F	GACGAGCTGTACAAGGGATCCGAGGCCGTGGAAC GCTTGC
	C765-R	TCCTTCACAAAGATCCTCTAGATTAAATGTCCTGT ATCTTGTTGATG
